# Fluid balance, change in serum creatinine and urine output as markers of acute kidney injury post cardiac surgery: an observational study

**DOI:** 10.1186/s40697-014-0019-4

**Published:** 2014-09-02

**Authors:** Katrina Chau, Travis Schisler, Lee Er, Dharmvir Jaswal, Christopher Cheung, Amanda Israel, John Bowering, Adeera Levin

**Affiliations:** Division of Nephrology, Faculty of Medicine, The University of British Columbia, St Paul’s Hospital, 1081 Burrard St, Vancouver, BC V6Z1Y6 Canada; Department of Anesthesiology, Pharmacology and Therapeutics, The University of British Columbia, St Paul’s Hospital, 1081 Burrard St, Vancouver, BC V6Z1Y6 Canada; Faculty of Medicine, The University of British Columbia, St Paul’s Hospital, 1081 Burrard St, Vancouver, BC V6Z1Y6 Canada

**Keywords:** Acute kidney injury, Diagnosis, Fluid resuscitation, Cardiac surgery

## Abstract

**Background:**

Acute kidney injury (AKI) is defined as oliguria or rise in serum creatinine but oliguria alone as a diagnostic criterion may over-diagnose AKI.

**Objectives:**

Given the association between fluid overload and AKI, we aimed to determine if positive fluid balance can complement the known parameters in assessing outcomes of AKI.

**Design:**

Prospective observational study.

**Setting:**

Teaching hospital in Vancouver, Canada.

**Patients:**

111 consecutive patients undergoing elective cardiac surgery from January to April 2012.

**Measurements:**

Outcomes of cardiac surgery intensive care unit (CSICU) and hospital length of stay (LOS) in relation to fluid balance, urine output and serum creatinine.

**Methods:**

All fluid input and output was recorded for 72 hours post-operatively. Positive fluid balance was defined as >6.5 cc/kg. Daily serum creatinine and hourly urine output were recorded and patients were defined as having AKI according to the AKIN criteria.

**Results:**

Of the patients who were oliguric, those with fluid overload trended towards longer LOS than those without fluid overload [CSICU LOS: 62 and 39 hours (unadjusted p-value 0.02, adjusted p-value 0.58); hospital LOS: 13 and 9 days (unadjusted p-value: 0.05, adjusted p-value: 0.16)]. Patients with oliguria who were fluid overloaded had similar LOS to patients with overt AKI (change in serum creatinine ≥ 26.5 µmol/L), [CSICU LOS: 62 and 69 hours (adjusted p value: 0.32) and hospital LOS: 13 and 14 days (adjusted p value: 0.19)]. Patients with oliguria regardless of fluid balance had longer CSICU LOS (adjusted p value: 0.001) and patients who were fluid overloaded in the absence of AKI had longer hospital LOS (adjusted p value: 0.02).

**Limitations:**

Single centre, small sample, LOS as outcome.

**Conclusions:**

Oliguria and positive fluid balance is associated with a trend towards longer LOS as compared to oliguria alone. Fluid balance may therefore be a useful marker of AKI, in addition to urine output and serum creatinine.

## Abrégé

**Contexte:**

L’insuffisance rénale aiguë (IRA) se définit comme une oligurie ou une élévation de la créatininémie. Par contre, l’oligurie comme unique critère diagnostique peut mener abusivement au diagnostic d’IRA.

**Objectifs:**

Étant donné l’association entre l’hyperhydratation et l’IRA, nous cherchons à déterminer si une balance liquidienne positive peut complémenter les paramètres connus dans l’évaluation des résultats de l’IRA.

**Type d’étude:**

Étude d'observation prospective.

**Contexte:**

Hôpital universitaire à Vancouver, Canada.

**Participants:**

111 patients consécutifs qui subissent une chirurgie cardiaque non urgente, entre janvier et avril 2012.

**Mesures:**

On a mis en parallèle les résultats de l’unité de soins intensifs en chirurgie cardiaque (USICC), de même que la durée de l’hospitalisation (soins actifs), avec la balance liquidienne, la diurèse et la créatininémie.

**Méthodes:**

On a mesuré les ingesta et excreta durant les 72 heures postopératoires. On a défini une balance liquidienne positive à >6,5 cc/kg. On a enregistré la créatininémie quotidienne et la diurèse aux heures, et on a déterminé que les patients souffraient d’IRA en nous basant sur les critères de l’Acute Kidney Injury Network (AKIN).

**Résultats:**

Parmi les patients oliguriques, ceux qui avaient une surcharge liquidienne tendaient davantage vers une hospitalisation prolongée que ceux qui n’en avaient pas [durée de soins actifs à l’USICC: 62 et 39 heures (valeur de p non ajustée: 0,02, valeur de p ajustée: 0,58); durée de soins actifs à l’hôpital: 13 et 9 jours (valeur de p non ajustée: 0,05, valeur de p ajustée: 0,16)]. Les patients présentant une oligurie qui présentaient aussi une surcharge liquidienne requéraient une durée de soins actifs similaire aux patients souffrant d’IRA (modification de la créatininémie ≥ 26,5 µmol/L), [soins actifs USICC: 62 et 69 heures (valeur de p ajustée: 0,32) soins actifs à l’hôpital: 13 et 14 jours (valeur de p ajustée: 0,19)]. Les patients présentant une oligurie, indépendamment de la balance liquidienne, bénéficiaient d’une durée de soins actifs à l’USICC prolongée (valeur p ajustée: 0,001), tandis que les patients en surcharge liquidienne, mais ne souffrant pas d’IRA bénéficiaient davantage de soins actifs à l’hôpital (valeur de p ajustée: 0,02).

**Limites de l’étude:**

Un seul centre, un échantillon restreint, les soins actifs considérés comme une issue.

**Conclusion:**

Les patients avec oligurie et une balance liquidienne positive ont nécessité des soins actifs prolongés à l’USICC, comparativement aux patients ne présentant qu’une oligurie. La balance liquidienne peut donc constituer un marqueur d’IRA, en plus de la diurèse et la créatininémie.

## What was known before

Oliguria as a sole diagnostic criterion may over-diagnose acute kidney injury.

## What this adds

Considering oliguria in the context of fluid status may be more useful than using oliguria as a sole diagnostic criterion. This study suggests that oliguria in the context of fluid overload identifies patients who require a longer length of stay as compared to patients who have oliguria alone.

## Background

Acute kidney injury (AKI) describes a spectrum of conditions ranging from a biochemical abnormality to oligoanuric renal failure requiring renal replacement therapy. It is associated with adverse short and long term outcomes in all populations studied, irrespective of the definition used. The definitions have varied over time in the published literature as well as in clinical practice. Recently published, standardized definitions from the KDIGO AKI Guideline [1] include the parameters of both serum creatinine and urine output. Consistency of definitions is important for clinical care and research across jurisdictions.

AKI as defined by the Acute Kidney Injury Network (AKIN) [2] is based on measurements of urine output or serum creatinine. According to AKIN, the urine output criterion for the diagnosis of AKI is a urine output ≤ 0.5 cc/kg/hour for greater than 6 hours. Changes in serum creatinine of as small as 26.5 µmol/L (or 0.3 mg/dl), are considered Stage 1 AKI within the AKIN classification. These small changes in serum creatinine have been consistently demonstrated to be associated with adverse outcomes, in numerous populations [3,4]. The AKIN classification has largely been adopted as the KDIGO AKI definition.

Although reduced urine output often precedes development of AKI as defined by a rise in serum creatinine, oliguria as a sole diagnostic criterion has been questioned by many as being too sensitive, and classifies too many people as having ‘AKI’ [5]. There is controversy as to whether reduced urine output alone is associated with adverse outcomes. Creatinine as a solitary biomarker for AKI itself has a multitude of well known flaws, and fluid overload may dilute creatinine concentration, thus potentially delaying the diagnosis of AKI.

Fluid balance or evidence of fluid overload has come to the attention of both clinicians and researchers as an alternative ‘biomarker’ for AKI. Fluid overload has been associated with adverse outcomes in critical care populations, and is often seen in association with AKI. There has been an ongoing ‘chicken and egg’ debate [6] regarding the role of fluid overload in AKI. It is unclear whether AKI is a consequence of fluid overload or rather that the presence of fluid overload in AKI indicates a more critically unwell patient requiring aggressive fluid resuscitation. Regardless, the association has been consistently demonstrated in the literature.

The current study sought to describe the individual parameters of fluid balance, urine output and changes in serum creatinine in varying combinations, in a population of patients undergoing elective cardiac surgery. We were interested in describing the constellations of these 3 parameters and their association with outcomes in this specific population.

The hypothesis of the study is that fluid balance is a useful parameter for the identification of patients - broadly diagnosed with AKI – who have worse outcomes.

## Methods

One hundred and eleven consecutive patients who were undergoing elective coronary artery bypass grafting (CABG), valve replacement or combined surgery over a 3-month period from January and April 2012 (convenience sample) were prospectively enrolled in this observational study. Patients on dialysis, those undergoing off pump surgical procedures, trans-catheter valve replacement surgery and isolated or combined thoracic aorta surgery were excluded.

Demographic and clinical baseline characteristics and risk factors were obtained from medical records and included: age, sex, weight, presence of diabetes, serum creatinine, eGFR (MDRD equation) <60 ml/min/1.73 m [2], exposure to intravenous contrast within two weeks of surgery and medications. Details of the surgical procedure were captured: duration of surgery (skin to skin), cardiopulmonary bypass (CPB) time and fluid administration totals from perfusionist records (type and amount of crystalloid and colloid), urine output, blood loss and blood transfusions.

Patients were observed and data tabulated for all fluid inputs and outputs for 72 hours post operatively, irrespective of location of the patient (critical care area or ward). Fluid balance was determined from all records upon discharge from the cardiac surgery intensive care unit (CSICU) and expressed as cc/kg. Daily serum creatinine and body weight was also recorded.

For the purposes of this study we defined AKI as per the AKIN criteria: the definition of AKI based on serum creatinine (AKI_Cr)_) was an increase from baseline of ≥26.5 µmol/L and the definition based on urine output (AKI_UO_) was urine output ≤ 0.5 cc/kg/hour for greater than 6 hours. We defined positive fluid balance for the purposes of this study as ≥ 6.5 cc/kg based on the rationale that insensible losses per day are approximately 500 cc/day. Thus, using the mean weight in the population (81 kg), we calculated that 6.5 cc/kg would approximate insensible losses (525 cc) over one day – the median CSICU length of stay – so that amounts over that would constitute a positive fluid balance.

Patients were categorized based on the combination of their statuses on the change in serum creatinine (≥26.5 µmol/L, denoted as SCr+/−), urine output (≤0.5 cc/kg/hr for greater than 6 hours, denoted as UO+/−) and fluid balance (≥6.5 cc/kg, denoted as FOL+/−). The combinations under consideration were: 1) SCr-, UO-, FOL- (no AKI), 2) SCr-, UO-, FOL+, 3) SCr-, UO+, FOL-, 4) SCr-, UO+, FOL+, and 5) SCr +.

The main outcomes of interest were duration of CSICU (hours/days) and hospital length of stay (LOS) (days).

### Statistical analysis

Continuous variables are presented as mean with standard deviation if normally distributed or median with interquartile range. Frequencies and percentages were presented for categorical variables.

The comparisons on the demographics, preoperative and intraoperative measures across groups were conducted using One-way ANOVA, Kruskal-Wallis test, Chi-square or Fisher’s exact tests where appropriate.

**Figure 1:**
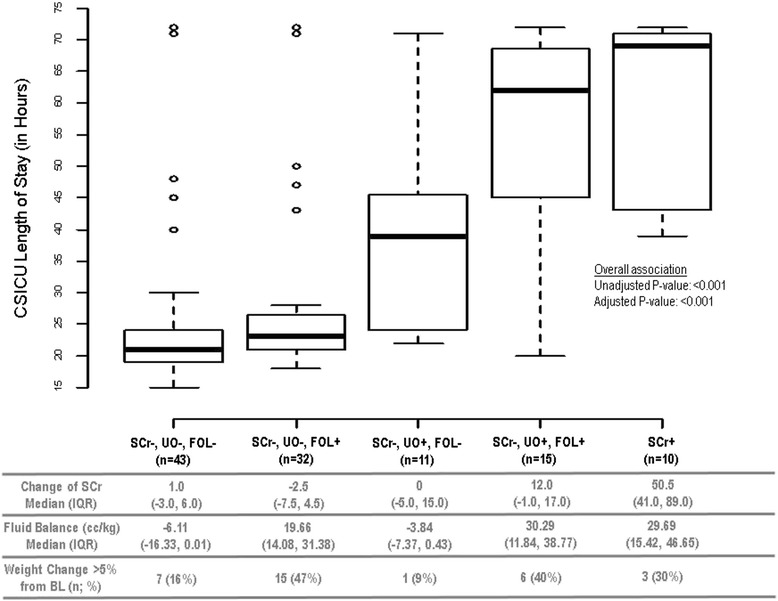
**Length of stay in cardiac surgery intensive care unit (CSICU), by categories of combinations of changes in serum creatinine, urine output and fluid overload.** *Abbreviations: SCr+: Meeting the criteria of Change in SCr ≥ 26.5 µmol/L ; SCr-: Not meeting the Change in SCr criteria. UO+: Meeting the criteria of Urine Output ≤0.5 cc/kg/hour for greater than 6 hours; UO-: Not meeting the Urine Output criteria. FOL+: Meeting the criteria of Fluid Overload, defined as fluid balance ≥ 6.5 cc/kg; FOL-: Not meeting the Fluid Overload criteria. IQR: Interquartile range. BL: Baseline.*

**Figure 2:**
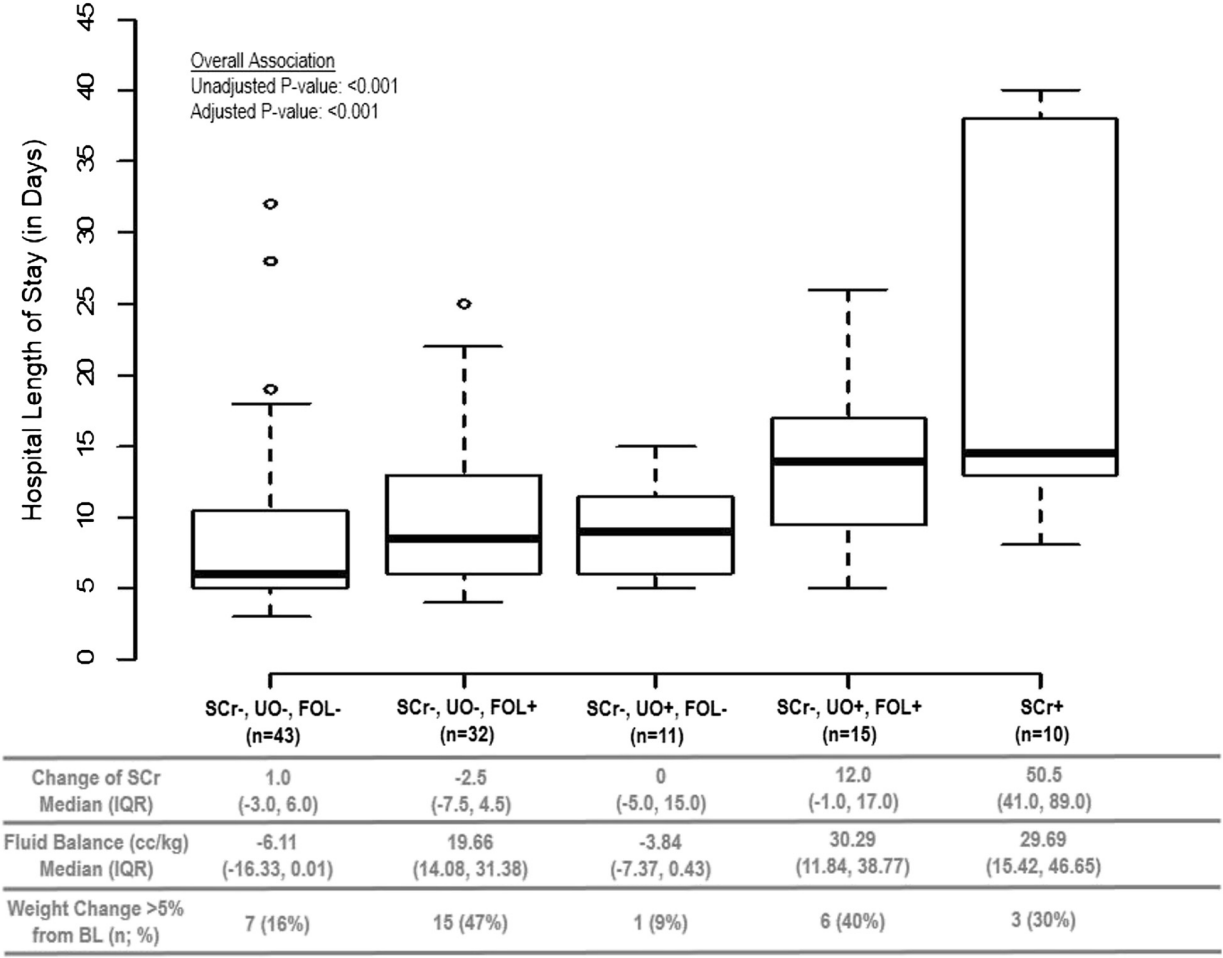
**Hospital length of stay by categories of combinations of changes in serum creatinine, urine output and fluid overload.** *Abbreviations: SCr+: Meeting the criteria of Change in SCr ≥ 26.5 µmol/L ; SCr-: Not meeting the Change in SCr criteria. UO+: Meeting the criteria of Urine Output ≤0.5 cc/kg/hour for greater than 6 hours; UO-: Not meeting the Urine Output criteria. FOL+: Meeting the criteria of Fluid Overload, defined as fluid balance ≥ 6.5 cc/kg; FOL-: Not meeting the Fluid Overload criteria. IQR: Interquartile range. BL: Baseline.*

The relationships between various combinations of change in serum creatinine/urine output/fluid balance and outcomes were examined by fitting two separate models, one for CSICU LOS and one for hospital LOS. Each LOS outcome was assumed to be log-normally distributed. We fitted a multiple linear regression model to examine the association between mean of log(LOS) and groups adjusting for potential confounding variables. The group variable (ie: combination of SCr/UO/FOL status) was parameterized [7] as in Table 1 for two reasons:

1. We wanted to treat the group variable as an ordinal variable as these group variables reflected severity as seen in Figures 1 and 2. By this parameterization, we could preserve the ordinal ranks without treating it as a continuous variable by arbitrarily assigning a numerical value to each category.
2. We could then compare between any two adjacent categories and hence obtain the best estimate of the increment/decrement (depending on whether the estimated parameter value was positive or negative) in the mean outcome between any two adjacent categories from the data. In other words, this would allow us to identify the ‘breakpoint(s)’ or ‘threshold(s)’ of the group variables at which significant changes occur in the outcome. For example, if only β_3_ (comparing group 3 and 4) was statistically significant, this would imply there were no differences in the mean log(LOS) in the first 3 groups and that furthermore, there was also no difference in the mean log(LOS) in the last 2 groups; hence group 4 was the critical breakpoint for an increase/decrease in the outcome.

**Table 1:** **Parameterization of the group variable in the multiple linear regression models**

	**Parameterization (corresponding parameter in the model)**			
**Group**	**X1 (β1)**	**X2 (β2)**	**X3 (β3)**	**X4 (β4)**
**1) SCr-, UO-, FOL-**	0	0	0	0
**2) SCr-, UO-, FOL+**	1	0	0	0
**3) SCr-, UO+, FOL-**	1	1	0	0
**4) SCr-, UO+, FOL+**	1	1	1	0
**5) SCr+**	1	1	1	1

Each group variable or combination of SCr/UO/FOL status was parameterized as above so that the group variable could be considered as an ordinal variable in the multiple linear regression model. β_1_ indicates a comparison between SCr-, UO-, FOL- and SCr-, UO-, FOL+, β_2_ indicates a comparison between SCr-, UO-, FOL + and SCr-, UO+, FOL- and so forth.

*SCr+: Meeting the criteria of Change in SCr ≥ 26.5 µmol/L; SCr -: Not meeting the Change in SCr criteria.*

*UO+: Meeting the criteria of Urine Output ≤0.5 cc/kg/hour for greater than 6 hours; UO-: Not meeting the Urine Output criteria.*

*FOL+: Meeting the criteria of Fluid Overload, defined as fluid balance ≥ 6.5 cc/kg; FOL-: Not meeting the Fluid Overload criteria.*

We adjusted for the covariates age, diabetes status, preoperative eGFR, duration of surgery, CPB time, blood transfusion and vasopressor requirement and percentage of patients with negative fluid balance during the intraoperative phase (only for CSICU LOS) in the models. These covariates were included based on clinical and statistical judgment on their relations to both group and outcome variables which resulted in the best fit to the data.

We considered a p-value of <0.05 as statistically significant. All statistical analysis was performed using SAS, version 9.3 (SAS institute, Cary, NC).

The study was approved by the institutional review board (Providence Health Care Research Ethics Board H11-03485) and conformed to the regulations of the institutional ethics committee on human research. Written consent was not required due to the observational nature of the study, and lack of interventions.

## Results

### Patient characteristics at baseline and peri-operatively

Table 2 describes the patient characteristics at baseline, preoperative and intraoperative measurements for the overall study cohort and by groups. Of the 111 patients, 76% were male, the mean age was 69 years and 28% were diabetic. The median serum creatinine was 85 μmol/L, corresponding to an eGFR of 72 ml/min/1.73 m [2], and 25% of patients had a pre-operative eGFR <60 ml/min. Over half the cohort received IV contrast within 2 weeks of the surgery, and 66% were receiving ACEi/ARB pre-operatively. 85% of the patients received a CABG or valve replacement surgery with the remainder having combined surgery.

**Table 2:** **Summary of patients’ characteristics at baseline, preoperative and intraoperative measures**

	**All (n = 111)**	**SCr-, UO-, FOL- (n = 43)**	**SCr-, UO-, FOL + (n = 32)**	**SCr-, UO+, FOL- (n = 11)**	**SCr-, UO+, FOL + (n = 15)**	**SCr + (n = 10)**	**P-value**
***Demographics***							
Age (years)	69 ± 10	69 ± 9	73 ± 8	60 ± 14	68 ± 11	69 ± 11	0.03
Male	84 (76%)	36 (84%)	21 (66%)	9 (82%)	11 (73%)	7 (70%)	0.43
Diabetes	31 (28%)	10 (23%)	4 (12%)	5 (45%)	6 (40%)	6 (60%)	0.01
***Preoperative Measures***							
Weight (kg)	80 (68, 91)	80 (71, 92)	69 (64, 84)	84 (78, 111)	85 (79, 93)	92 (68, 107)	0.01
Height (cm)	170 ± 10	170 ± 10	169 ± 11	172 ± 8	171 ± 10	170 ± 12	0.94
Creatinine (μmol/L)	85 (71, 103)	87 (69, 101)	80 (71, 93)	81 (70, 103)	88 (70, 96)	117 (90, 144)	0.01
eGFR (mL/min/1.73 m2)	72 ± 17	77 ± 23	73 ± 19	81 ± 23	63 ± 28	52 ± 18	0.01
eGFR <60 mL/min/1.73 m2	28 (25%)	8 (19%)	7 (23%)	2 (18%)	4 (27%)	7 (70%)	0.03
Recent IV Contrast	57 (51%)	15 (35%)	21 (64%)	5 (45%)	9 (60%)	7 (70%)	0.05
Pre-op ACE/ARB	73 (66%)	27 (63%)	20 (62%)	9 (82%)	11 (73%)	6 (60%)	0.72
Pre-op Diuretics	31 (28%)	11 (25%)	6 (19%)	3 (27%)	5 (33%)	6 (60%)	0.16
***Surgery Type***							
MVR Only/AVR Only	35 (32%)	12 (28%)	13 (41%)	2 (18%)	4 (27%)	4 (40%)	0.17
CABG Only	59 (53%)	28 (65%)	13 (41%)	8 (73%)	6 (40%)	4 (40%)	
CABG + MVR/AVR	17 (15%)	3 (7%)	6 (18%)	1 (9%)	5 (33%)	2 (20%)	
***Intraoperative Measures***							
Duration of Surgery (mins)	177 (146, 209)	188 (150, 209)	156 (122, 190)	176 (117, 200)	186 (151, 233)	200 (173, 243)	0.007
CPB Time (mins)	92 (72, 108)	92 (77, 105)	75 (62, 96)	84 (46, 100)	103 (88, 148)	103 (95, 140)	0.01
Intraoperative pRBC	24 (22%)	6 (12%)	9 (25%)	1 (0%)	7 (40%)	6 (50%)	0.007
Intraoperative Crystalloid (cc)	1260 (875, 1727)	1200 (900, 1780)	1350 (900, 1663)	850 (700, 1260)	1050 (845, 1650)	1386 (1000, 1880)	0.43
Intraoperative Colloid (cc)	500 (500, 500)	500 (500, 650)	500 (500, 600)	500 (500, 500)	500 (500, 500)	500 (500, 500)	0.54
Total Fluid Administered (cc)	1850 (1485, 2380)	1820 (1500, 2380)	1901 (1715, 2398)	1350 (1100, 1960)	1730 (1520, 2404)	2165 (1600, 3412)	0.08
Total Urine Output (cc)	250 (160, 400)	325 (175, 500)	250 (187, 470)	220 (150, 350)	260 (125, 400)	125 (70, 225)	0.07
Total Fluid Balance (cc)	129 (846, 1700)	1350 (900, 1720)	1147 (875, 1519)	1240 (515, 1825)	1200 (−686, 1650)	1765 (105, 2003)	0.68
Negative Fluid Balance at Completion of Surgery	8 (7%)	1 (2%)	0 (0%)	0 (0%)	5 (33%)	2 (20%)	<0.001
***Postoperative Medications***							
Vasopressors	85 (77%)	29 (67%)	27 (84%)	6 (54%)	13 (87%)	10 (100%)	0.04
Number of Vasopressors	1 (1, 2)	1 (0, 1)	1.5 (1, 2)	1 (0, 2)	2 (1, 2)	2.5 (2, 4)	<0.001

Vasopressors used were: Adrenaline, noradrenaline, dobutamine, milirinone and vasopressin.

*SCr+: Meeting the criteria of Change in SCr ≥ 26.5 µmol/L ; SCr -: Not meeting the Change in SCr criteria.*

*UO+: Meeting the criteria of Urine Output ≤0.5 cc/kg/hour for greater than 6 hours; UO-: Not meeting the Urine Output criteria.*

*FOL+: Meeting the criteria of Fluid Overload, defined as fluid balance ≥ 6.5 cc/kg; FOL-: Not meeting the Fluid Overload criteria.*

*Continuous variables are presented in mean ± standard deviation or median with interquartile range. Categorical variables are presented in count with percentage. One-way ANOVA or Kruskal-Wallis test were used to compare among groups for the continuous variables; Chi-Square test or Fisher’s Exact test were used for the categorical variables.*

*eGFR was calculated using the MDRD equation.*

The median duration of surgery was 177 minutes with a median CPB time of 92 minutes. 22% received intraoperative blood transfusion, and the median total fluid administered intraoperatively was 1850 cc. At the end of surgery, the median total fluid balance measured was positive 1269 cc, while 7% of patients had a negative balance at completion. Intraoperative urine output was less than 500 cc in all groups.

### AKI by urine output and serum creatinine criteria and relationship to fluid balance

**Figure 3:**
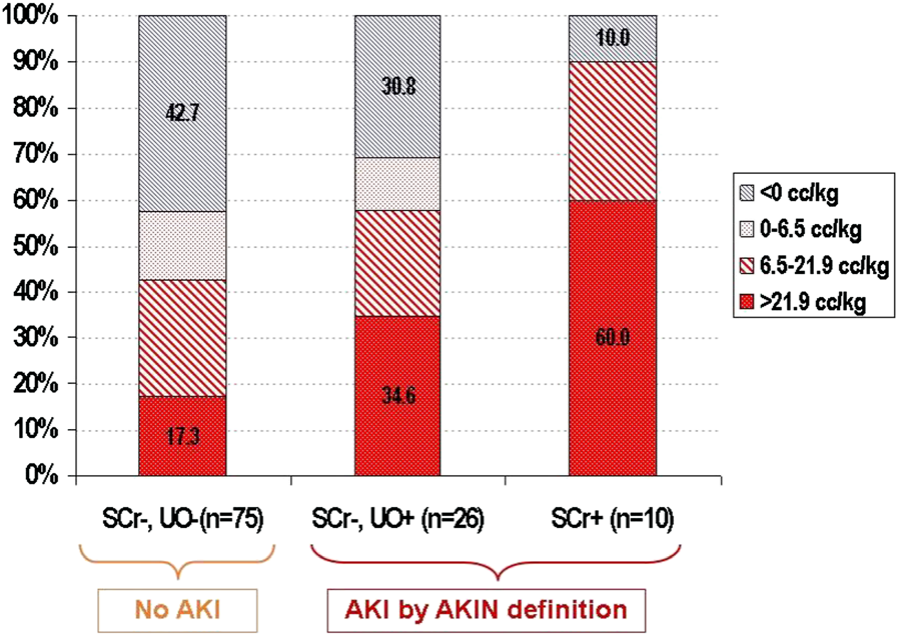
**Fluid balance as categorized by AKIN criteria for acute kidney injury.** *Abbreviations: AKIN: Acute Kidney Injury Network diagnostic criteria for AKI* [2]*. SCr+: Meeting the criteria of Change in SCr ≥ 26.5 µmol/L; SCr-: Not meeting the Change in SCr criteria. UO+: Meeting the criteria of Urine Output ≤0.5 cc/kg/hour for greater than 6 hours; UO-: Not meeting the Urine Output criteria.*

Using the accepted definitions of AKI, the incidence of AKI_Cr_ was 9% (n = 10) and AKI_UO_ was 31% (n = 35). Of those with AKI_Cr_, 9/10 also met urine output criteria. Figure 3 describes the amount of positive fluid balance in patients with SCr-/UO- (no AKI), SCr-/UO + (AKI_UO_) and SCr + (AKI_Cr_). Note that those with AKI_Cr_ had the largest proportion of positive fluid balance (>6.5 cc/kg), and those with AKI by either definition had larger proportion of positive fluid balance than those who did not have AKI using either parameter.

### Outcomes

#### Length of stay in CSICU

The median intensive care unit LOS was 24 (IQR: 21, 47) hours.

Figure 1 depicts the distribution of LOS in CSICU in association with the five combinations of changes in serum creatinine, urine output and fluid overload. Note that those with no change in serum creatinine and no reduced urine output (irrespective of fluid overload) [ie: 1) SCr-, UO-, FOL- and 2) SCr-, UO-, FOL+)] had the shortest LOS in CSICU (medians of 21–23 hours). Among those with oliguria and no change in serum creatinine [ie: 3) SCr-, UO+, FOL- and 4) SCr-, UO+, FOL+], those with fluid overload had longer LOS in CSICU (median = 62 hours) compared to those without (median = 39 hours). Those with no change in serum creatinine but with oliguria and positive fluid balance had similarly long LOS in the CSICU as those who simply had elevated serum creatinine [ie: 5) SCr+] (medians of 62 and 69 hours, respectively).

Table 3 provides the parameter estimates from the multiple linear regression models. The unadjusted analyses indicated that there were two comparisons of patient groups in which there was a significant increase in mean LOS in CSICU. Firstly when patients had oliguria alone (SCr-, UO+, FOL-) compared to patients with fluid overload alone (SCr-, UO-, FOL+) the estimated increase in mean log(LOS) was 0.31 [95% C.I.: 0.04, 0.57; p-value = 0.02]. Secondly when comparing patients with oliguria *and* fluid overload (SCr-, UO+, FOL+) to those with oliguria alone (SCr-, UO+, FOL-), there was an estimated increase in mean log(LOS) of 0.35 [95% C.I.: 0.05, 0.65; p-value = 0.02]. In the adjusted analyses, the only difference remained was between those with and without oliguria (estimated increase in mean log(LOS): 0.44 [95% C.I.: 0.18, 0.70]; p-value = 0.001).

**Table 3:** **Estimates of the group variable parameters resulted from the multiple linear regression models**

**Outcome**	**Parameter**	**Unadjusted model**			**Adjusted model**		
		**Estimate**	**95% C.I.**	**p-value**	**Estimate**	**95% C.I.**	**p-value**
**CSICU Length of Stay**	**β1 (SCr-, UO-, FOL + vs. SCr-, UO-, FOL-)**	0.09	−0.08, 0.26	0.31	0.12	−0.05, 0.29	0.16
	**β2 (SCr-, UO+, FOL- vs. SCr-, UO-, FOL+)**	0.31	0.04, 0.57	0.02	0.44	0.18, 0.70	0.001
	**β3 (SCr-, UO+, FOL + vs. SCr-, UO+, FOL-)**	0.35	0.05, 0.65	0.02	0.08	−0.21, 0.37	0.58
	**β4 (SCr + vs. SCr-, UO+, FOL+)**	0.16	−0.15, 0.46	0.31	0.14	−0.14, 0.41	0.32
**Hospital Length of Stay**	**β1 (SCr-, UO-, FOL + vs. SCr-, UO-, FOL-)**	0.18	−0.06, 0.41	0.14	0.29	0.04, 0.54	0.02
	**β2 (SCr-, UO+, FOL- vs. SCr-, UO-, FOL+)**	−0.05	−0.40, 0.30	0.78	−0.13	−0.52, 0.25	0.49
	**β3 (SCr-, UO+, FOL + vs. SCr-, UO+, FOL-)**	0.39	−0.004, 0.79	0.05	0.30	−0.12, 0.67	0.16
	**β4 (SCr + vs. SCr-, UO+, FOL+)**	0.36	−0.05, 0.77	0.08	0.27	−0.14, 0.67	0.19

*SCr+: Meeting the criteria of Change in SCr ≥ 26.5 µmol/L; SCr -: Not meeting the Change in SCr criteria.*

*UO+: Meeting the criteria of Urine Output ≤0.5 cc/kg/hour for greater than 6 hours; UO-: Not meeting the Urine Output criteria.*

*FOL+: Meeting the criteria of Fluid Overload, defined as fluid balance ≥ 6.5 cc/kg; FOL-: Not meeting the Fluid Overload criteria.*

#### Length of stay in hospital

The median hospital LOS for the population was 9 (IQR: 6, 14) days.

Figure 2 depicts the relationship between the LOS in hospital with various combinations of change in serum creatinine, urine output and fluid overload. Of the patients who were oliguric with no change in serum creatinine [ie: 3) SCr-, UO+, FOL- and 4) SCr-, UO+, FOL+] those who were fluid overloaded had longer hospital LOS (median = 13 days) as opposed to those who were not fluid overloaded (median = 9 days). Note that patients were who oliguric and fluid overloaded but with no change in serum creatinine [ie: 4) SCr-, UO+, FOL+] had similarly long length hospital LOS as those who simply had elevated serum creatinine [ie: 5) SCr+] (medians of 13 and 14 days, respectively).

In the unadjusted analyses (see Table 3), we found two group comparisons which were marginally associated with longer LOS. There was a significant increase in the mean log(LOS) when comparing oliguric patients with fluid overload (SCr-, UO+, FOL+) and without fluid overload (SCr-, UO+, FOL-): the estimated increase was 0.39 [95% C.I.: −0.004, 0.79; p-value = 0.05]. There was also some suggestion of an increase in the mean log(LOS) in patients with AKI_Cr_ when compared to oliguric patients with fluid overload (SCr-, UO+, FOL+): the estimated increase was 0.36 [95% C.I.: −0.05, 0.77; p-value = 0.08].

In the adjusted analyses (also see Table 3), the only critical breakpoint in an increase in mean log(LOS) was when we compared patients who had no AKI and were not fluid overloaded (SCr-, UO-, FOL-) to those who had no AKI but were fluid overloaded: the estimated increase was 0.29 [95% C.I.: 0.04, 0.54; p-value = 0.02].

The change in serum creatinine, fluid balance and weight gain in each of the different categories is described in the lower panel of Figures 1 and 2. Those with highest weight gain had more positive fluid balance. Patients who were oliguric but not fluid overloaded were in mild negative balance (SCr-, UO+, FOL-; median −3.8 cc/kg) as were patients who did not have AKI (median −6.1 cc/kg). Patients that were oliguric and fluid overloaded (SCr-, UO+, FOL+) had the same degree of fluid overload as those patients that had AKI_Cr_ with a median positive fluid balance of 30 cc/kg. Given the average weight of the patients (81 kg), 30 cc/kg equates to approximately 2 litres, after accounting for insensible losses.

## Discussion

This study describes the outcomes for patients undergoing elective cardiac surgery who develop AKI, using various definition parameters. We used an integrated categorization of AKI that incorporates both the AKIN criteria and fluid balance. We wanted to demonstrate the merits of considering each parameter - serum creatinine, urine output and fluid balance - together, as they are associated with the relevant outcomes of CSICU and hospital LOS. Our data suggests a potential difference in LOS for cardiac surgery patients with AKI as defined using these different parameters in various constellations. In those patients with oliguria, there was an increased duration of CSICU LOS, but those with oliguria *and* fluid overload had trends towards longer LOS in both CSICU and in hospital. The value of ‘contextualizing’ urine output by including a measure of overall fluid balance has not been overtly discussed in any of the recent guideline proposals.

Since oliguria as a sole diagnostic criterion for the diagnosis of AKI has been considered oversensitive, we suggest that in the context of fluid overload it may in fact portend worse outcomes (defined here as longer LOS). Some publications suggest that the current criterion of ≤0.5 cc/kg/hour for greater than 6 hours may identify patients who are at no greater risk of mortality or need for dialysis [8,9]. This analysis reaffirms that oliguria is still a marker of risk that should be taken seriously, but needs to be contextualized within the clinical situation, and fluid balance measurements. This is consistent with other data [10]. Given that weight is a sensitive indicator of positive fluid balance, this may allow practical measurements in non critical care settings to be used.

Although AKI_UO_ is imperfect as only a small number of patients with AKI_UO_ subsequently develop AKI_Cr_ [11], reduction in urine output below thresholds may serve to alert clinicians as the ‘canary in the coal mine’ [12]. Use of more stringent criteria may lead to delayed recognition of early AKI. While at the current time there are limited renoprotective strategies, as newer agents become available, it will be important to diagnose AKI as early as possible. A study examining the timing of nephrology referral and outcomes suggested that delayed nephrology consultation is associated with worse prognosis [13] in hospitalized patients.

Concealed AKI as a result of dilution of creatinine by volume expansion has been recognized [14-16] as a potential diagnostic pitfall when relying on serum creatinine changes. Fluid overload increases the time to identification of an increase in creatinine [15] and misses cases altogether [17]. In this study, patients with oliguria and fluid overload did have smaller changes in serum creatinine levels. The potentially important (median) rise in serum creatinine of 12 μmol/L (IQR: −1, 17) did not meet the threshold of 26.5 μmol/L, but within the context of known dilution this small increase may be indicative of rises of serum creatinine within the threshold range. The data in our study suggest that oliguria in combination with fluid overload may point towards worse outcomes than oliguria alone. This may be in part because fluid overload masks a true rise in creatinine.

The mechanism by which fluid overload may cause increased mortality is multifactorial [18]. Intravenous fluid administered, whether colloid or crystalloid, does not stay within the vascular space in critically ill patients [19]. Interstitial lung oedema leads to requirement for mechanical ventilation. Increased cardiac preload secondary to fluid overload may lead to cardiac dysfunction. Specific to the kidney, elevated renal venous pressure can impair kidney perfusion and increase interstitial pressure. In our study, patients with fluid overload even without AKI by either diagnostic criteria had significantly longer hospital LOS, suggesting that fluid overload in and of itself may be harmful in addition to perpetuating existing injury. Of note, the ‘adverse’ outcomes in our study occurred in those with approximately 2 litres positive balance in 24 hours. This is consistent with findings in the SOAP study, where patients had a mean positive daily fluid balance of 1 to 1.5 litres.

Patients in our study with either AKI_UO_ or AKI_Cr_ had more positive fluid balance than those without AKI, again consistent with other studies [20,21]. Fluid overload is associated with complications [22] of cardiac surgery, failure to recover from AKI [23] and mortality [21,24]. We did not capture those outcomes in this study.

There are limitations to this study. This is a single centre study, using a convenience sample of consecutive patients undergoing elective surgery. The outcomes are limited to short term (CSICU and hospital LOS), and not to 30 day mortality or peri-operative complications, as in other studies. However, the purpose of the study was to describe AKI according to the different criteria, and determine if those definitions had relevance to LOS. It is important to acknowledge that prolonged LOS is an important outcome from an individual patient and health care utilization perspective. We did not capture the rationale for fluid administration so the issue of cause and effect are not easy to disentangle from these data. This was an observational study and there were differences in across the 5 categories of fluid balance, urine output and serum creatinine. However, in the adjusted models, these were taken into account. As a descriptive study, these data serve to catalogue current practice in post cardiac surgery patients and allow generation of testable hypotheses.

The strengths of this study are the careful tabulation of all input and output from the time of surgery until 72 hours post operatively; that patients appeared to receive similar amounts of volume within the operative period, and had similar urine outputs. Thus, all of the differences that occurred with fluid administration took place after similar intraoperative volume resuscitation, which may permit the development of interventions to be tested in the postoperative period.

There is accumulating literature on the benefits of restrictive fluid management strategies [25,26] in critically ill patients. It may be prudent to trial similar strategies in this highly vulnerable cardiac surgery population. A balance must be struck between the importance of perioperative fluid resuscitation and the potential adverse effect of post-operative fluid overload. In this study, of the 8 patients in negative balance following surgery, 7 had AKI_UO_ with fluid overload or AKI_Cr_.

## Conclusions

Our study has an important practical message. Attention to fluid balance, with and without perturbations in serum creatinine or oliguria, may help to more accurately identify those patients with AKI at risk of adverse outcomes. Our data suggest that oliguric patients with fluid overload may have worse outcomes than those with oliguria alone. The definition and recognition of AKI is evolving. It is important that our approach to fluid management, resuscitation and limitations in fluid accumulation similarly evolve.
